# The placenta shed from goats with classical scrapie is infectious to goat kids and lambs

**DOI:** 10.1099/vir.0.000151

**Published:** 2015-08

**Authors:** David A. Schneider, Sally A. Madsen-Bouterse, Dongyue Zhuang, Thomas C. Truscott, Rohana P. Dassanayake, Katherine I. O'Rourke

**Affiliations:** ^1^​ Animal Disease Research Unit, Agricultural Research Service, US Department of Agriculture, Pullman, WA, 99164-6630, USA; ^2^​ Department of Veterinary Microbiology and Pathology, College of Veterinary Medicine, Washington State University, Pullman, WA, 99164-7040, USA

## Abstract

The placenta of domestic sheep plays a key role in horizontal transmission of classical scrapie. Domestic goats are frequently raised with sheep and are susceptible to classical scrapie, yet potential routes of transmission from goats to sheep are not fully defined. Sparse accumulation of disease-associated prion protein in cotyledons casts doubt about the role of the goat's placenta. Thus, relevant to mixed-herd management and scrapie-eradication efforts worldwide, we determined if the goat's placenta contains prions orally infectious to goat kids and lambs. A pooled cotyledon homogenate, prepared from the shed placenta of a goat with naturally acquired classical scrapie disease, was used to orally inoculate scrapie-naı¨ve prion genotype-matched goat kids and scrapie-susceptible lambs raised separately in a scrapie-free environment. Transmission was detected in all four goats and in two of four sheep, which importantly identifies the goat's placenta as a risk for horizontal transmission to sheep and other goats.

The transmissible spongiform encephalopathies (TSE) are a heterogeneous group of disorders differing in aetiology, pathology, host range, strain repertoire and efficient transmission routes. The transmissible agent, the prion, is unique amongst infectious disorders in that it is widely believed to principally consist of a misfolded protease-resistant isoform of a host-encoded protein, the prion protein (PrP) (Caughey *et al.*, 2009; Soto, 2011). Classical scrapie is a prion disease of domestic sheep (*Ovis aries*) that causes significant economic burden to sheep industries worldwide. Scrapie eradication programmes are largely based on the observation that classical scrapie is efficiently transmitted through contact with the placenta shed by infected ewes (Pattison *et al.*, 1972) and that susceptibility is limited by polymorphisms in the prion protein gene, *PRNP* (Goldmann, 2008). Eradication programmes focused on such factors have resulted in dramatic decreases in disease prevalence, but eventual eradication could be delayed or infection reintroduced if other reservoirs of scrapie prions are not identified and managed.

Classical scrapie also affects domestic goats (*Capra hircus*) (González *et al.*, 2010; Hadlow *et al.*, 1980; Konold *et al.*, 2010; Pattison & Millson, 1961) but much less is known about its pathology, including potential routes of transmission to sheep and other goats. In sheep, the shed placenta contains abundant accumulation of disease-associated misfolded prion protein (PrP^Sc^) (Andréoletti *et al.*, 2002; Lacroux *et al.*, 2007; Tuo *et al.*, 2001, 2002), is infectious (Onodera *et al.*, 1993; Pattison *et al.*, 1972; 1974; Race *et al.*, 1998), plays a key role in horizontal transmission (Hoinville *et al.*, 2010; Touzeau *et al.*, 2006) and contributes to environmental contamination (Andréoletti *et al.*, 2002; Gough & Maddison, 2010). Since sheep and goats are sometimes co-housed during parturition, and since goats have been used as surrogate dams for orphaned lambs, one of the most likely scenarios for scrapie transmission from goats to sheep is during the post-partum period through oral exposure to parturient material. Given the sparse accumulation of PrP^Sc^ in the placenta of goats as compared to sheep (O'Rourke *et al.*, 2011), intra- and interspecies transmission by this route is not certain. The primary purpose of this experiment was to determine if classical scrapie in goats (caprine scrapie) is orally transmissible to other goats and sheep via the placenta.

All animal use and care was approved by the Washington State University Institutional Animal Care and Use Committee. The experiment was conducted using a previously described source of tissue (O'Rourke *et al.*, 2011). In brief, the placenta donor goat (3950; Nubian) originated from a naturally infected US goat herd [herd 2, O'Rourke *et al.* (2011)] reported to be without direct contact with sheep for at least 5 years and located on premises without prior history of scrapie disease. At the time of regulatory investigation, goat 3950 was not clinical but was determined to be scrapie-infected through antemortem rectal biopsy testing and standard scrapie immunohistochemistry (IHC). The goat was acquired at 20 months of age and by 34 months of age began showing clinical signs of classical scrapie disease including progressive weight loss, truncal scratching and ataxia. At 35 months of age, the goat gave birth to three kids and the placenta (G797) was immediately collected for processing. The donor goat was euthanized at 38 months of age for humane reasons associated with progression of scrapie disease. All three kids associated with this placenta developed clinical scrapie disease by 2.5 years of age. Additionally, the donor goat and all three kids had subclinical infection with small ruminant lentivirus (SRLV) as determined by repeated serological testing as conducted by the Washington Animal Disease Diagnostic Laboratory (Pullman, WA, USA) using competitive inhibition ELISA (Small Ruminant Lentivirus Antibody Test kit, cELISA; VMRD) (Herrmann *et al.*, 2003a, b). The *PRNP* genotypes of all animals in this study were determined by DNA sequence analysis as previously described (O'Rourke *et al.*, 2011). Donor goat 3950 and all three kids associated with this placenta were heterozygous for the central caprine *PRNP* haplotypes 1 and 2 (White *et al.*, 2008), which only differ at codon 240 [respectively, proline (P) and serine (S)]. Both haplotypes are associated with susceptibility to classical scrapie disease (Vaccari *et al.*, 2009).

All cotyledons from one fetal unit of placenta G797 were pooled, stored for 2 years at − 80 °C, and then homogenized just days prior to use as inoculum. Pooled cotyledons (116 g total wet weight) were homogenized in a new Oster blender using the setting ‘mix’, first for 5 min neat and then as a final 83 % (w/v) homogenate in PBS for 2 min. Aliquots (∼4 ml each) of the G797 cotyledon homogenate were briefly stored at − 20 °C before use. As previously described (O'Rourke *et al.*, 2011), accumulation of disease-associated PrP in G797 cotyledon homogenate was determined by Western blot analysis using mAb F99/97.6.1 and by scrapie ELISA (HerdChek CWD Ag Test; IDEXX Laboratories). As seen by Western blot (Fig. 1a), typical proteinase K-resistant PrP (PrP^res^) bands were readily detected in the obex hindbrain of donor goat 3950 (ob, lane 1; loading 450 μg tissue wet weight equivalent) and in a sodium phosphotungstic acid (Na-PTA) extract of 90 mg tissue wet weight equivalent of G797 cotyledon homogenate (cot, lane 2). No PrP^res^ bands were observed in a Na-PTA extract of 90 mg tissue wet weight equivalent of a similarly prepared cotyledon homogenate derived from the shed placenta of scrapie-unexposed goat 4113 (Fig. 1a: cot, lane 3); this goat and cotyledon homogenate were also heterozygous for caprine *PRNP* haplotypes 1 and 2. Determination of PrP^Sc^ content in the obex and G797 cotyledons from goat 3950 was by scrapie ELISA using twofold serial dilutions of neat homogenates (Fig. 1b). The tissue equivalents loaded into each assay well were expressed in terms of total protein (BCA Protein Assay kit; Pierce Biotechnology). As determined by linear regression (adjusted R^2^ >0.98) and interpolation (at the background-corrected optical density of 0.75), the PrP^Sc^ content of G797 cotyledons were ∼183-fold less than that present in this goat's obex. These results are in agreement with the relatively sparse accumulation previously reported for the placentas of other naturally infected goats and other fetal units from this donor goat's placenta (O'Rourke *et al.*, 2011).

**Fig. 1.**
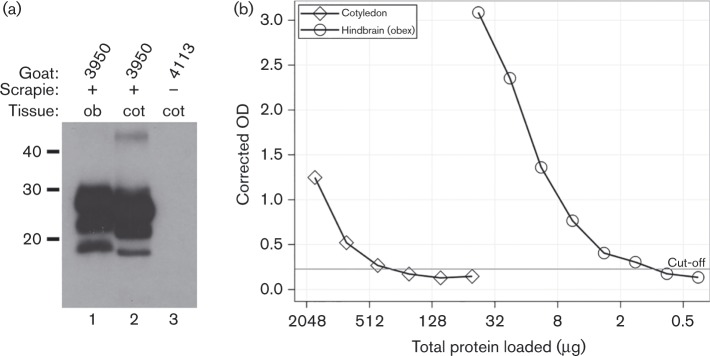
Comparison of the disease-associated prion protein content in the hindbrain at the level of the obex and in shed cotyledons from donor goat 3950. (a) Proteinase K-resistant prion protein (PrP^res^) bands were detected by Western blot analysis in the obex (ob, lane 1) and cotyledon (cot, lane 2; Na-PTA precipitate) homogenates derived from the scrapie-infected donor goat 3950 but not in similarly prepared *PRNP* genotype-matched cotyledon homogenate from the scrapie-naïve donor goat 4113 (cot, lane 3; Na-PTA precipitate). (b) Scrapie ELISA titrations of disease-associated prion protein (PrP^Sc^) in shed cotyledons (open diamonds) and obex (open circles) from donor goat 3950. PrP^Sc^ content is expressed as background-corrected arbitrary units of optical density (corrected OD); tissue equivalents loaded at each twofold serial dilution are expressed in terms of total protein content (*x*-axis scaling, log_2_). As determined per manufacturer's directions, the scrapie ELISA cut-off sensitivity was 0.228 and is shown as a horizontal line.

Recipient goats consisted of four Saanen kids born to scrapie-unexposed does. One goat kid was homozygous for caprine *PRNP* haplotype 1 (4471); the other three were heterozygous for haplotypes 1 and 2. Recipient sheep consisted of four white- or mottled-faced lambs born to scrapie-unexposed ewes. Recipient lambs were homozygous for the scrapie-susceptible ovine *PRNP* haplotype, which codes for valine at position 136 (i.e. VV_136_) (Goldmann, 2008). Kids and lambs were born in a holding facility in which scrapie-infected animals had never been housed. Newborn animals nursed colostrum for 48 h and were then moved and raised by hand in scrapie-unexposed isolation rooms, one for kids and one for lambs. Newborns were inoculated at 48–72 h of age via the oral route by placing a single, partially thawed aliquot of G797 cotyledon homogenate (∼3.3 g wet tissue weight equivalent) near the back of the tongue, immediately after which kids nursed a bottle of fresh cow's milk and lambs nursed a bottle of lamb's artificial milk replacer. Kids and lambs were later weaned onto a balanced ration of grass and alfalfa hay with access to appropriate mineral supplements. Since these animals were raised indoors, each received subcutaneous injections of 75 000 IU vitamin D3 at approximately three-week intervals (Vitamin A D injection; Agri Laboratories).

As depicted in a timeline (Fig. 2a), scrapie infection status was monitored antemortem by biopsy of the rectoanal mucosa-associated lymphoid tissue (RAMALT) (González *et al.*, 2005) and in sheep, also by biopsy of the nictitating membrane (biopsies 3 and 4) (O'Rourke *et al.*, 2000). Scrapie IHC using monoclonal antibody F99/97.6.1 was applied to formaldehyde-fixed, paraffin-embedded tissues as previously described (O'Rourke *et al.*, 2011). Antemortem lymphoid accumulation of PrP^Sc^ was detected in three of four recipient goats but in only one (sheep 4442) of four recipient sheep (Fig. 2a, 2b). One recipient sheep (4440) was euthanized at 747 days post-inoculation (p.i.) due to development of an abomasal emptying disorder. Although PrP^Sc^ was not detected by scrapie IHC in the obex or in any of the lymphoid tissues examined from this sheep (Table 1, example shown in Fig. 2b), PrP^res^ accumulation was evident by Western blot analysis after Na-PTA extraction of retropharyngeal and ileocecal lymph node homogenates (Fig. 2c, lanes 2 and 5). Recipient goat 4474 was euthanized at 784 days p.i. for comparison with recipient sheep 4440. Similar to sheep 4440, antemortem accumulation of PrP^Sc^ had not been detected in goat 4474 (last biopsy at 721 days p.i.; Fig. 2b). In contrast to sheep 4440, PrP^Sc^ and PrP^res^ were readily detected in multiple post-mortem tissues of recipient goat 4474 by 784 days p.i. (Table 1), though still not in the RAMALT. One recipient sheep (4442) and three recipient goats (4470, 4471 and 4479) were eventually removed from isolation to await development of clinical signs. As summarized in Table 1, transmission of scrapie infection was confirmed in four of four recipient goats and in two of four recipient sheep.

**Fig. 2.**
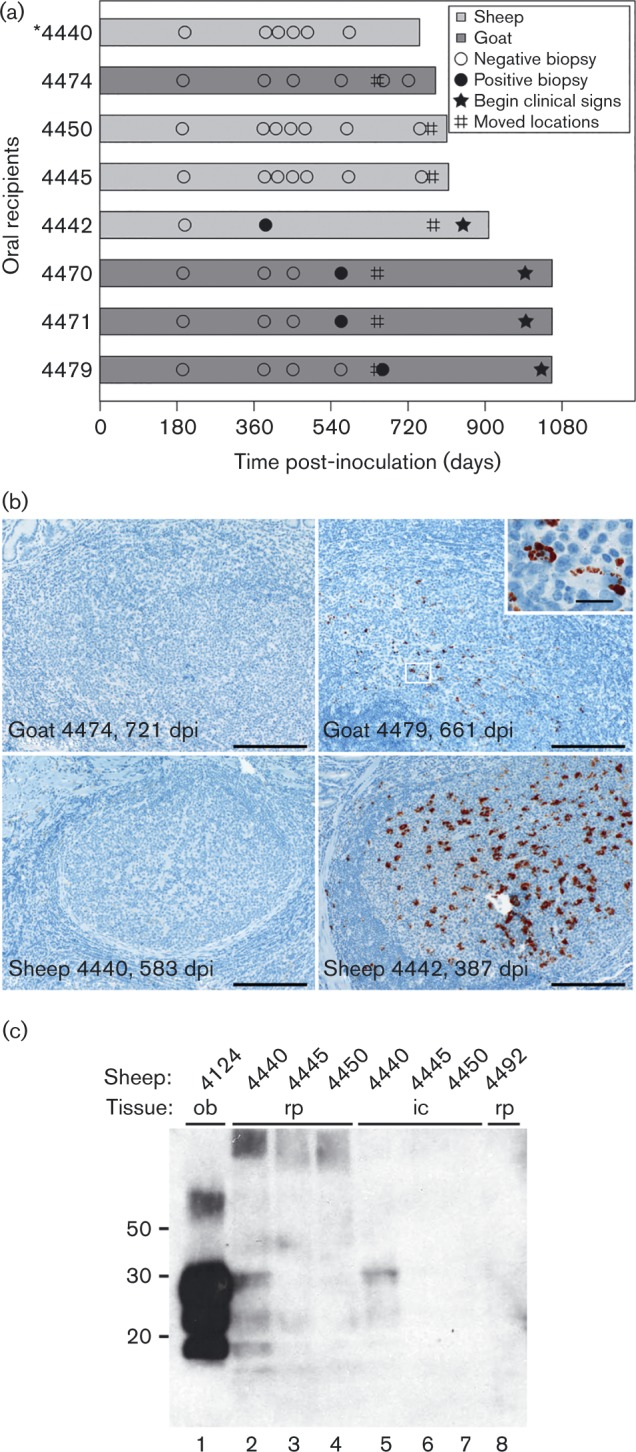
Detection of classical scrapie transmission in recipient goat kids and lambs. (a) Timeline for recipients orally inoculated with pooled cotyledon homogenate derived from a scrapie-infected goat. Shown are the results of biopsies (circles; open = PrP^Sc^ not detected, closed = PrP^Sc^ detected), movement from isolation to a quarantine farm environment (#), start of clinical signs of scrapie (solid star) and post-mortem examination (end of bar) of each recipient animal. *Sheep 4440 euthanized due to intercurrent disease (abomasal emptying disorder). (b) Representative images of scrapie IHC (PrP^Sc^ = red chromagen deposits) on antemortem biopsies of the rectoanal mucosa-associated lymphoid tissue. Images in which PrP^Sc^ was not detected (left column) or was detected (right column) are presented for each recipient species (goat, top row; sheep, bottom row). Age at time of biopsy is given as days post-inoculation (dpi). Accumulation of PrP^Sc^ is relatively sparse in goat 4479 as compared with sheep 4442. A higher power inset (bar = 20 μm) of the region of interest (white box) is provided for goat 4479. All other bars = 200 μm. (c) Detection of proteinase K-resistant prion protein (PrP^res^) bands by Western blot after Na-PTA extraction of retropharyngeal (rp) and ileocecal (ic) lymph node homogenates. The typical three PrP^res^ glycoforms are only evident in the lymph nodes of recipient sheep 4440 (rp, lane 2; ic, lane 5) but not recipient sheep 4445 (lanes 3 and 6) or 4450 (lanes 4 and 7). Assay controls included the Na-PTA extractions of a dilute scrapie-positive obex homogenate (positive control: ob, lane 1) and of a retropharyngeal lymph node from a scrapie uninfected sheep (negative control: rp, lane 8).

**Table 1.** Summary of results for goats and sheep orally inoculated as neonates with homogenate prepared from a placenta shed from a goat with clinical scrapie disease IHC, immunohistochemistry; na, not applicable since animal euthanized without evidence of clinical disease; nd, PrP^d^ not detected by immunoassay; POS, PrP^d^ detected by immunoassay; PrP^d^, disease-associated prion protein; SUS, suspect and limited chromagen deposits; WB, Western blot; –, not determined.

Animal	Time to PrP^d^ detection	Time to clinical disease	Time to post-mortem	Post-mortem immunoassay					
	(days p.i.)	(days p.i.)	examination	Obex	RPLN		ICLN		RAMALT
			(days p.i.)	IHC	IHC	WB	IHC	WB	IHC
**Goats**									
4474	784	na	784	POS	POS	POS	POS	POS	SUS
4470	564	994	1057	POS	POS	–	POS	–	POS
4471*	564	995	1057	POS	POS	–	POS	–	POS
4479	661	1032	1056	POS	POS	–	POS	–	POS
**Sheep**									
4440	747	na	747†	nd	nd	[POS]‡	nd	[POS]	–
4450	ND	na	811	nd	nd	[nd]	nd	[nd]	–
4445	ND	na	815	nd	nd	[nd]	nd	[nd]	–
4442	387	849	908	POS	POS	–	POS	–	POS

* Goat 4471 was homozygous for the caprine *PRNP* haplotype 1. The other three goats were heterozygous for haplotypes 1 and 2.

† Euthanized due to inter-current disease (abomasal emptying disorder).

‡ [Bracketed] result is for WB after Na-PTA extraction; corresponding standard WB result = nd.

The genotypes of goats used in this study included caprine *PRNP* haplotypes 1 and 2 (White *et al.*, 2008), which differ in sequence only at codon 240. The mature PrP produced by haplotypes 1 and 2 are identical, however, since several C-terminal amino acids, including codon 240, are removed during post-translational maturation of the protein (Stahl *et al.*, 1990). Thus, goats in this study all expressed the archetypal PrP of sheep and goats (referred to as ARQ), which codes for alanine (A) at codon 136, arginine (R) at codon 154 and glutamine (Q) at codon 171 (Goldmann, 2008). Transmission of scrapie to all four goat recipients indicates a placental prion titre high enough to efficiently infect ARQ/ARQ goat kids by the oral route. The recipient sheep used in this study were VRQ/VRQ, a genotype known to be at greatest risk for developing scrapie under field conditions (Baylis *et al.*, 2004). However, a recent oral inoculation study in sheep demonstrates that transmission from an ARQ/ARQ donor is efficient in PrP homologous (i.e. ARQ/ARQ) recipients but results in significantly prolonged incubation in heterologous (ARQ/VRQ or VRQ/VRQ) recipients (González *et al.*, 2012). Similarly, oral transmission of cattle-origin bovine spongiform encephalopathy prions is reduced in VRQ/VRQ sheep as compared with ARQ/ARQ sheep (McGovern *et al.*, 2015; Tan *et al.*, 2012). These findings may explain why transmission of caprine scrapie was only confirmed in two of four VRQ/VRQ sheep recipients in this study but, given constraints that limited the incubation time available for study, transmission to the other two sheep cannot be ruled out. Nonetheless, these studies collectively suggest an equal or greater risk of transmission to ARQ/ARQ sheep.

It is unknown if co-infection of the donor goat with an SRLV had a confounding influence on the outcome of this experiment. Small ruminant lentiviruses are a highly related group of retroviruses with potential for interspecies transmission (Leroux *et al.*, 2010), causing persistent infections that can result in several types of chronic inflammatory diseases (Blacklaws, 2012). Co-infection of sheep and goats with classical scrapie and an SRLV increases the peripheral distribution of PrP^Sc^ (González *et al.*, 2010; Salazar *et al.*, 2010) and the infectious titre of prions in the milk of ewes with SRLV-associated mastitis (Lacroux *et al.*, 2008). In this study, direct effects on the recipient kids and lambs are unlikely since SRLV infection was not detected by 16 months of age. Although effects on the donor goat cannot be ruled out, the increased distribution of PrP^Sc^ that has been reported in co-infected animals was only in association with viral pathology in the respiratory tract or mammary gland; similar lesions are not reported in the placenta. Nevertheless, very little is known about the mechanisms underlying PrP^Sc^ accumulation at the placental feto-maternal interface (Alverson *et al.*, 2006; Andréoletti *et al.*, 2002; Lacroux *et al.*, 2007; Tuo *et al.*, 2001, 2002) or the basic mechanisms underlying enhanced cellular accumulation of PrP^Sc^ associated with SRLV co-infection (Stanton *et al.*, 2008).

In conclusion, this study importantly demonstrates that the placenta of goats infected with classical scrapie can transmit scrapie to susceptible goat kids and lambs via a natural route of exposure despite relatively sparse accumulation of PrP^Sc^ within the goat's placenta. Thus, like for sheep, the parturient materials and post-partum period of goats must be considered transmission risks for other susceptible small ruminants and environmental contamination.
